# Significant association between Taq1 gene polymorphism in vitamin D receptor and chronic spontaneous urticaria in the Northeast of Iran

**DOI:** 10.1186/s12948-021-00145-w

**Published:** 2021-05-27

**Authors:** Maryam Khoshkhui, Farzaneh Iravani, Farahzad Jabbari-Azad, Hadi Zare Marzouni, Jalil Tavakkol-Afshari, Hanieh Zamani, Maryam Davarpanah, Alireza Hamidian Jahromi, Mojgan Mohammadi

**Affiliations:** 1 Allergy Research Center, Mashhad University of Medical Sciences, Mashhad, Iran; 2 Genetic Research Center, Mashhad University of Medical Sciences, Mashhad, Iran; 3 Qaen School of Nursing and Midwifery, Birjand University of Medical Sciences, Birjand, Iran; 4 Immunology Research Center, Mashhad University of Medical Sciences, Mashhad, Iran; 5 Plastic and Reconstructive Surgery Department, Rush University Medical Center, Chicago, IL USA

**Keywords:** Taq1 gene polymorphism, Vitamin D receptor, Chronic spontaneous urticaria, Allergy, CSU, VDR gene, Taq1

## Abstract

**Objective:**

Chronic spontaneous urticaria (CSU) is defined as urticaria with an unknown etiology which persists for more than 6 weeks. CSU is an uncomfortable cutaneous condition that occurs due to an immune-mediated inflammatory reaction. Many studies have demonstrated that vitamin D deficiency and single-nucleotide polymorphisms in the vitamin D receptor (VDR) impact the immune response. In the current study, the frequency of the Taq1 polymorphism in the VDR gene were compared between patients with CSU and individuals without CSU.

**Methods:**

In a case–control study, a group of CSU patients (n = 100) was compared with a group of healthy age- and gender-matched individuals as a control group (n =100) who visited our center between 2015 and 2017. After DNA extraction from EDTA-containing blood, polymerase chain reaction (PCR–RFLP) was used to determine the presence of the Taq1 polymorphism. Serum vitamin D levels were measured using ELISA method (Abcam, Cambridge, USA).

**Results:**

Genotyping for Taq1 polymorphism showed that TT, Tt and tt genes frequency in the CSU group were 36%, 54%, and 10% respectively. The TT, Tt and tt genotypes had a distribution of 50%, 47% and 3% respectively in the control group. The mean serum vitamin D level in the CSU group was 19.88 ± 8.14 ng/ml, which was not significantly correlated with the Taq1 polymorphism (P = 0.841). There was a significant relationship between Taq1 gene polymorphism (tt genotype) and CSU (P = 0.038). Tt genotype increased the risk of CSU (odds ratio = 1.596), and inheritance of tt genotype increased the risk even further (odds ratio = 4.630).

**Conclusion:**

The frequency of Taq1 genotype polymorphism in the VDR gene was significantly higher in patients with CSU compared to the control group. The tt genotype polymorphism may be a risk factor for CSU.

## Introduction

Urticaria is one of the most common skin diseases, in which patients can present with a variety of symptoms, including the sudden development of pruritic lesions or angioedema. Urticaria is classified as acute or chronic based on the duration of symptoms. Chronic urticaria (CU) is diagnosed when a disease has been continuously or intermittently present for at least 6 weeks. The chronic and acute forms of urticaria differ in etiology, pathophysiology, and underlying mechanism [1]. Chronic urticaria accounts for 5–20% of all urticaria cases, and the cause remains unknown in 70–90% of patients. The prevalence of chronic urticaria showed significant regional differences [2, 3]. Chronic urticaria has a significant impact on quality-of-life due to the symptom of constant itching [1]. Chronic urticaria has been divided into two subgroups by multiple societies including the European Academy of Allergy and Clinical Immunology (EAACI), Global Allergy and Asthma European Network (GA^2^ LEN), European Dermatology Forum (EDF), and World Allergy Organization (WAO): chronic spontaneous urticaria (CSU) and chronic inducible urticaria [4, 5].

The burden of CU for patients, their family and friends, the healthcare system, and society is substantial, thus understanding its underlying pathogenesis is important [5, 6]. The close interaction between the neuroendocrine and immune systems is the core of several inflammatory disorders of the skin, including CSU [7]. Numerous theories have been proposed to explain the pathogenesis of CSU; yet, none have been proven to thoroughly explain the mechanism of the disease [8].

A potential role for vitamin D in the underlying pathogenesis of allergic diseases has received increased attention in recent decades. A possible role for vitamin D in the treatment of immune-mediated conditions, including autoimmune diseases, has also been investigated [9]. As atopic dermatitis, chronic urticaria, and allergic contact dermatitis all involve immune dysregulation, the role of vitamin D in these three common chronic skin disorders has begun to be explored [10].

The biologically active form of vitamin D known as (1, 25)-dihydroxyvitamin D3, or vitamin D3, is the key regulator of calcium and phosphorus metabolism. Vitamin D3 plays an important role in cell differentiation, apoptosis, intrinsic immunity, differentiation of monocytes into macrophages, and modulation of macrophage response through inhibition of cytokines and chemokines production. Vitamin D effectively inhibits the proliferation of lymphocytes and increases the secretion of cytokines including gamma interferon, interleukin-2, and interleukin-12 [11–18].

Vitamin D is hydrophobic; as such, it must be bound to carrier proteins for transportation in the blood. The major carrier is called group-specific component or vitamin D-binding protein (VDBP). While the half-life of its precursor, 25-hydroxycholecalciferol, is several weeks, vitamin D3 has a short reported half-life of a few hours [19].

The vitamin D receptor (VDR) is encoded by the VDR gene, which is located on chromosome 12 (12q14). Mutation in the functional regions of the VDR gene can have profound impacts on vitamin D function in the body. The VDR is also known as NR1I1 (nuclear receptor subfamily 1, group I, member 1), and it is a member of the nuclear receptor family of transcription factors [20]. Binding of Vitamin D to its ligand leads to alteration of the expression of specific genes in certain tissues [17].

The gene encoding the receptor of vitamin D is located on the long arm of chromosome 12 (12q13.11). It has 11 exons and a length of about 6.5 kb. Several single-nucleotide polymorphisms (SNPs) have been reported in various exons of the VDR gene. Most of these SNPs occur in the 3' region, which is evaluated by restriction enzymes i.e., Taq1, ApaI and BsmI. Specific polymorphisms (such as ApaI, EcoRV, Tru9I, Taq1, BsmI, and FokI) have been identified in different parts of the VDR gene. Except for FokI, which is located on exon 2, the others are located between exon 8 and exon 9 [21]. Several studies have examined the association between the VDR gene polymorphisms and various diseases, including rheumatoid arthritis, diabetes, and cancer [13–16, 22].

However, the relationship between any polymorphism and CSU has not been studied before. Therefore, in the current study, we investigated the association of Taq1 polymorphism within the VDR gene in patients with CSU compared with healthy individuals (without CSU). We also investigated the relationship between the Taq1 polymorphism in VDR gene and serum levels of vitamin D in CSU patients.

## Materials and methods

### Patients and controls

This case–control study was conducted at the Ghaem Hospital of Mashhad University of Medical Sciences and the Booali Research Center, in Mashhad, Iran, between 2015 and 2017. Patients at the allergy clinic were referred to the Immunology Laboratory of Booali Institute after a diagnosis of CSU from an allergy specialist. After obtaining consent, blood and serum samples were taken from patients with CSU to examine the Taq1 polymorphism in the vitamin D receptor gene and to measure serum vitamin D levels. For comparison, samples were collected from a group of healthy blood donors (control group) after they completed a standard health questionnaire and provided consent for participation in the present research project.

A total of 10 ml of whole blood was sampled from each subject in both control and CSU groups in four tubes. Two tubes were used for serum separation to measure the serum vitamin D level. The sera were stored at − 70 °C until the test was performed. Additional tubes of blood were used to determine the genotype of Taq1 polymorphism in the vitamin D receptor gene. DNA extraction was performed using a PCR blood extraction kit (YektaTajhiz Azma Co).

### Determine the serum levels of vitamin D

In this study, blood samples were taken from individuals to determine the serum level of vitamin D. Serum was separated from the blood samples by centrifugation for 15 min at 2500 RPM. Measurement of serum vitamin D level was performed by ELISA method using Human Vitamin D ELISA kit (Abcam, Cambridge, USA). Serum samples were collected according to the manufacturer's instructions.

### Statistical analysis

Statistical analysis was performed using SPSS software (version 16). Characteristics of the subjects, including distribution frequencies, central and descriptive indicators, and percentages, were expressed using descriptive statistical methods. Also, for comparison between the CSU and the control groups, inferential statistical methods including the Kolmogorov–Smirnov test, Levene's test for equality of variances, Chi-Square test, Fisher's exact test, t-test, Mann–Whitney test, and logistic regression analysis were applied.

In this study, individual’s samples were examined for both clinical and genotypes of Taq1 polymorphism in the VDR gene as well as serum vitamin D also levels of vitamin D serum. The Kolmogorov–Smirnov statistical test confirmed the normal distribution of the quantitative data for further statistical evaluations (P > 0.05). As such the above mentioned so parametric statistical tests were used for the data analysis in this study.

## Results

Table 1 shows the demographic characteristics of the participants in the CSU and control groups. There were no significant differences between the two groups in terms of age (P = 0.977) or gender (P = 0.631).

**Table 1:** Demographic data of the subjects in two groups of patients: CSU (case group) and the control group (healthy individuals)

Characteristics	CSU	Control	P-value
**Age (years)**^a^	34.76 ± 12.11	34.72 ± 7.33	0.977
**Gender**			
M	28 (28%)	25 (25%)	0.631
F	72 (72%)	75 (75%)	
**Marital status**			
Single	28 (28%)	–	**–**
Married	72 (72%)		
**Education**			
Illiterate	7 (7%)	–	**–**
Student	6 (6%)		
Third grade middle school	23 (23%)		
Diploma	20 (20%)		
Associate diploma Degree	7 (7%)		
BSc	27 (27%)		
MSc	10 (10%)		
PhD	0		
**Confronting anxiety**	57 (57%)	–	**–**
**Taking medication**	35 (35%)	–	**–**
**Alcohol consumption**	0	0	**–**
**Smoking**	0	0	**–**
**Illicit drug use**	0	0	**–**
Total	100 (100%)	100 (100%)	–

M: Male; F: Female; CSU: Chronic spontaneous urticaria; BSc: Bachler degree or equivalent; MSc: Master degree or equivalent; PhD: Doctorate degree or equivalent

^a^Data are presented as mean ± standard deviation

The frequency of genotypes in the CSU group and control group is shown in Table 2. The results of genotype data analysis with the Pearson Chi-Square test showed a significant difference between frequency of genotypes in the CSU and control groups (P = 0.038).

**Table 2:** Frequency of Taq1 polymorphism genotype present in the vitamin D receptor (VDR) gene in the plasma samples from both case (patients with CSU) and the control groups (healthy individuals)

Groups	Genotype			P-value
	TT	Tt	tt	
**Case**				
Number	36	54	10	0.038
Percentage	36%	54%	10%	
**Control**				
Number	50	47	3	
Percentage	50%	47%	3%	

To confirm the results of genetic studies for both genotypes of Taq1 polymorphisms in the VDR gene, Hardy–Weinberg analysis was performed. The percentage of genotypes obtained and  Q-squared in acceptable levels with percentage showed that genotypes do not match the Hardy–Weinberg equation (P < 0.05). Therefore, the deviation from the equilibrium in this sampling was considered to be significant and the populations under the study was not in equilibrium.

### Taq1 polymorphism in the VDR gene

Data analysis by ANOVA test indicated that there is a significant relationship between Taq1 polymorphism in the VDR gene and CSU (P = 0.038). The presence of t allele in Taq1 polymorphism of the VDR gene was found to be a risk factor for  CSU. The Tt genotype increases the risk of CSU (odds ratio = 1.596), and the tt genotype increases the risk even further (odds ratio = 4.630) (Table 3).

**Table 3:** Frequency of genotypes, risk chances and 95% CI based on the genotypes of Taq1 polymorphism in the vitamin D receptor (VDR) gene

Group	Case		Control		OR	95% CI	P-value
	Number	Percentage	Number	Percentage			
TT	36	36	50	50	1	Reference	0.038
Tt	54	54	47	47	1.596	2.850–0.893	
tt	10	10	3	3	4.630	18.028–1.189	

OR: Odds Ratio; CI: Confidence Interval; Case group: individuals with CSU; Control group: healthy individuals

### Serum levels of vitamin D

**Fig. 1:**
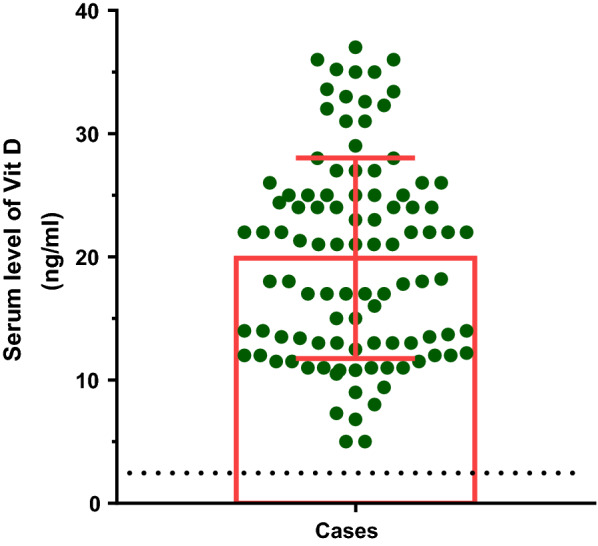
Serum levels of vitamin D in the case (patients with CSU) group (ng/ml)

The mean serum level of vitamin D (25-hydroxyvitamin D) in the CSU group was 19.88 ± 8.14 ng /ml (Fig. 1).

### Taq1 polymorphism in the VDR gene and level of vitamin D

**Fig. 2:**
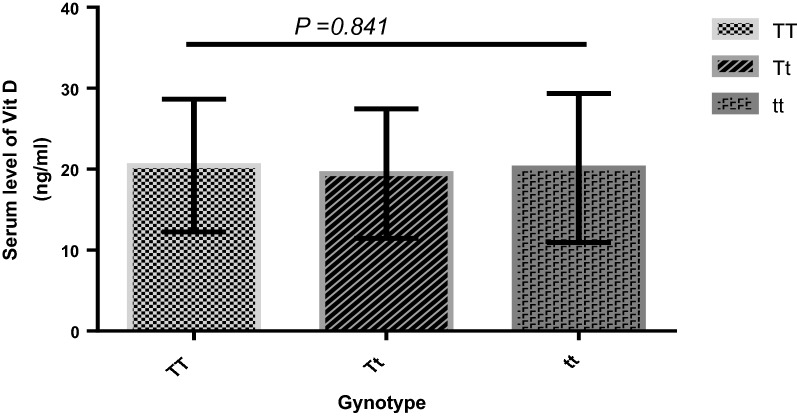
Mean serum levels of vitamin D (ng/μl) and its correlation with Taq1 polymorphism in the vitamin D receptor (VDR) gene in patients with CSU

Statistical analysis using the ANOVA test showed no significant relationship between mean serum levels of vitamin D and genotypes of Taq1 polymorphism in the VDR gene (P > 0.841). The mean serum concentrations of vitamin D in CSU patient blood samples were 20.46 ± 8.24 ng/μl for the TT genotype (n = 36), 19.44 ± 8.00 ng/μl for the Tt genotype (n = 54), and 20.16 ± 9.2 in ng/μl for the tt genotype (n = 10) (Fig. 2).

## Discussion

This results of this study demonstrated that the Taq1 polymorphism in the VDR gene may be one of the genetic factors implicated in the development of CSU. Specifically, the inheritance of the t allele in the Taq1 polymorphism of the VDR gene, as opposed to the T-allele seems to be a risk factor for CSU. The role of vitamin D in the immune system and immune responses has been explored in many studies [11, 16]. The role of this vitamin in immune regulation was first theorized after the identification of vitamin D receptors in lymphocytes. Prior studies have shown that the active form of vitamin D (1,25-dihydroxy-vitamin D3) has a direct effect on active and naïve T helper cells, regulatory T cells, activated B cells, and dendritic cells [23].

The interaction of vitamin D with the immune system is the important factor that activates both inherent and acquired immune systems. In recent years, with increasing knowledge about the effects of vitamin D, researchers have studied its influence in a variety of diseases and different kinds of cancer [24, 25]. The data indicates that vitamin D is involved in the regulation of immune responses for both innate and adaptive immunity. Vitamin D has a major role in the innate immune system in response of pathogenic organisms and tissue damage. Natural killer (NK) cell function depends on adequate vitamin D levels. There are some in vitro studies which have shown that vitamin D suppress NK cell activity and IFN-γ secretion [26, 27]. However, little is known about the in vivo effect of vitamin D on NK cell function.

Many studies have described the relationship between vitamin D and immune-related diseases, including allergy [28]. A study by Nasiri et al. (2018) investigated the role of vitamin D receptor gene polymorphisms, including BsmI (rs1544410) and Fok1 (rs2228570) in CSU. The authors demonstrated that vitamin D levels in patients with CSU were lower than those in the control group. There was also a significant association between vitamin D level and CSU activity. The authors concluded that this polymorphism might be a potential risk factor for CSU and that changes in the pathway of vitamin D at the gene or protein level might be a risk factor for the development of CSU [29].

Another study was performed by Perez et al. in 2017 which indicated that there was a significant difference between serum levels of vitamin D among the two groups of case and controls. The serum levels of vitamin D in CSU patients were significantly lower than control group [30]. In another study by Boonpiyathad et al., the authors examined the serum vitamin D levels in CSU patients and similarly found that vitamin D levels were significantly lower in the case group compared to the control individuals. There are some studies that show a negative correlation between serum level of vitamin D and IgE and IL-4 i.e., Ozkaraet et al. The results of these studies indicate that vitamin D deficiency is common in patients with CU [31]. The cause of vitamin D deficiency in CSU patients is likely multifactorial. One of the factors could be the presence of other polymorphisms that affect gene function. For example, gene on gene interactions are effective in the response of a gene to the production of other genes. Ethnic differences are one of the most influential factors in genetic studies. A recent study in China demonstrated the relationship of the VDR gene FokI (rs2228570) polymorphism and the incidence of CSU in the Chinese Han population. Unlike our study, the authors did not find a significant relation between Taq1 polymorphism and CSU. This result is not surprising, as there were significant genetic disparities between populations [32]. In alignment with our results, they did not report a significant relationship between serum vitamin D levels in subjects with CSU with Taq1 polymorphism in the VDR gene.

## Conclusion

 The results of the current study demonstrated that there was a relationship between a CSU diagnosis and Taq1 polymorphism, but there was no significant relationship between serum vitamin D levels in CSU patients and Taq1 polymorphism in VDR gene. Further studies with larger numbers of patients are required to investigate the exact relationships between the severities of CSU symptoms and vitamin D serum levels. Further functional studies are also required to clarify the mechanism of Taq1 polymorphism affecting CSU and the effect of VDR gene polymorphism on the risk of developing CSU.

## Data Availability

All materials and data are available to publication.
